# The Timber Supply

**Published:** 1882-11

**Authors:** 


### CENSUS BULLETIN FOR TEXAS, FLORIDA, ALABAMA, MISSISSIPPI, AND MINNESOTA.

The Census Office has commenced to issue a series of bulletins regarding the timber supply of the United States, the investigation being conducted by Professor C. S. Sargent, of Harvard University. Five of these have recently appeared, relating to the States of Texas, Florida, Alabama, Mississippi, and Minnesota, each bulletin treating of a State, and each accompanied by a map of the State, colored to represent the regions covered by different species of valuable timber, and those from which the timber has been cut.

The pine supply of Texas appears to be confined to the eastern part, bordering upon Louisiana, and is entirely comprised in forty-two counties. The total amount standing on May 31, 1880, is estimated at 67,508,500,000 feet (board measure), composed of the three species known as long-leaved pine (*Pinus Australis*), short-leaved pine (*Pinus mitis*), and loblolly pine (*Pinus faeda*), in about the proportions of twenty, twenty-six, and twenty-one. The cut during the year ending on the above date was 274,440,000 feet, or about four-tenths of one per cent. of the total amount standing.

The pine supply of Florida appears to be quite uniformly distributed over the State. The long-leaved pine covers western Florida and the upper half of the peninsula. The timber of the lower half of the peninsula is almost entirely pitch-pine (*Pinus cubensis*). This is found also everywhere in the neighborhood of the coast, especially that of the Atlantic. The amount of the long-leaved pine standing is estimated at 6,615,000,000 feet, and the cut during the census year at 208,054,000 feet, or a little more than three per cent. of the total amount. It is noted that the "long-leaved pine forests of Central and Southern Florida are less heavy than those of the northern part of the State, and south of latitude 29 degrees N. are of little present commercial value, although included in the above estimate."

The southern fourth of Alabama is covered with forests of the long-leaved pine, mixed in the northern part with much hard wood. A comparatively narrow belt of pine runs nearly across the State between latitudes 32 degrees and 33 degrees, besides several smaller bodies farther north. The total amount of standing pine is estimated at 21,192,000,000 feet, and the cut during the census year 245,396,000 feet, being a little more than one per cent. of the total supply.

The pine region of Mississippi is the southeast two-fifths of the State. From this main body a strip of pine, mixed with hard wood, extends northward, east of the middle line of the State, nearly to the northern boundary, following in general the divide between the Big Black and the Tombigbee rivers. A small body also exists in the northeastern corner of the State. The total amount of standing pine in the State is estimated at 23,975,000,000 feet, of which 17,200,000 feet are long-leaved pine, the remainder being of the short-leaved species. The cut was 115,775,000 feet, or about one-half of one per cent. of the total supply. It is to be noted in regard to the above States that the annual cut is very small, varying from a small fraction of one per cent. up to about three per cent. only. The color upon the maps, representing the total cut areas, shows that only a small proportion of the timber has been used as yet—the color being confined mainly to narrow belts along the streams. This is in decided contrast to the conditions in the Northern States.

The forest region of Minneseta may be roughly defined as the northeastern half of the State. This is bordered on the south and west by hard wood, while northward the pine forests gradually change in character and decrease in value. Lakes and swamps cover large areas, while the pines become small and scattered, and cease entirely before reaching the northern boundary of the State, their place being taken by tamarack and cedar. The species of pine is that known as white pine (*Pinus strobus*). Among the hard woods the principal species are oaks, sugar maple, and poplar. The total amount of standing white pine is estimated at 6,100,000,000 feet, while 540,977,000 feet, or 8.6 per cent., were cut during the census year. The amount of standing hard woods is estimated as 57,600,000 cords. The cut for the census year, exclusive of 7,825,-000 staves and 547,000 sets of headings, was 36,884,000 feet. The map shows that nearly one-half of the total area of the pine lands has already been cut. In the older States of Michigan and Wisconsin the proportion is even greater.

This is, we believe, the first systematic attempt ever made to estimate our timber resources, and the success with which Professor Sargent is meeting in dealing with this difficult subject reflects the highest credit upon him and his assistants.

---

A VISITOR, on calling at a friend's house during the session of the Legislature, was questioned thus by a little boy : "Where is your axe?" "What do you mean, little boy?" asked the visitor. "I heard pa say that the reason you came to town was because you had an axe to grind."

